# An Account of a General Inoculation at Painswick

**Published:** 1786

**Authors:** J. C. Jenner

**Affiliations:** Surgeon, at Painswick, in Gloucestershire


### X. *An Account of a general Inoculation at Painswick. By* the fame.

IN the month of May, 1785, the inhabitants of this town and its environs were alarmed by the appearance of the fmall-pox, which, notwithftanding every precaution ufed to prevent it from fpreading, raged with great violence, and deftroyed nearly one third of thofe who were infected by it. This fatality may, in fome meafure, perhaps, be attributed to a contagious fever and epidemic ague which prevailed at the fame time, and to the heat of the atmofphere. Under thefe circumftances, inoculation was propofed as the moft likely means of putting a ftop to the fatal effects of the difeafe.

On the 26th of May I began with the poor, and by the latter end of July following the number of inoculated patients, on my lift, amounted to feven hundred and thirty-eight. Of this number only two died; and the deaths even of thefe could not properly be afcribed to the fmall-pox: one of thefe patients, a young man, twenty-two years old, had, for feveral months, laboured under an intermittent, which had brought on a general anafarca. I inoculated him at his own earneft defire; but no inflammation flammation took place on his arm, and he died a few days after, without any ſymptom of ſmall-pox. The other patient was a child, three months old, who had about thirty puſtules, and appeared to be going on well till a few days after the eruption, when he was ſuddenly ſeized with a convulſive fit, (a complaint to which he had frequently been ſubject from his birth) and died.

Of the ſeven hundred and thirty-ſix who recovered, many had been afflicted with intermittents of ſeveral months ſtanding, attended with anaſarcous ſwellings; others were recovering from the malignant fever: ſeveral of the patients were lying-in women, and their children; others were children labouring under dentition; and many were perſons afflicted with phthiſis pulmonalis, ſcrophula, &c. &c. Two caſes only of confluent ſmall-pox appeared: one of theſe was a girl of a ſcrophulous habit; the other a woman, very much reduced by the malignant fever. Many women were inoculated during the early ſtages of pregnancy, without any ill conſequence, and ſome in the later months: four inſtances of the latter I ſhall here mention, in addition to thoſe inſerted in the London Medical Journal, Vol. V. page 399.

CASE

### CASE I.

Jane Parker, aged twenty-feven, was inoculated May 25, 1785, being then in the eighth month of pregnancy, and on the 1ft of June the eruptive fever commenced. The eruptions (few in number) appeared on the third day. She recovered; and went about her bufinefs as ufual. On the 18th of July fhe felt fymptoms that convinced me the child was dead; and on the 23d fhe was delivered of a dead child, with about thirty large puftules on its body, the bafes of which were in a gangrenous ftate.

### CASE II.

Mary Ellis, aged forty-two, in the ninth month of pregnancy, was inoculated May 25, 1785. On the 1ft of June the eruptive fever came on, together with pains, refembling thofe of labour. A laxative medicine was given; and afterwards an opiate, which relieved her. She had few eruptions, and did well. On the 10th of June fhe fell down ftairs: this accident brought on labour, and fhe was delivered of a dead child, which had no appearance of eruption on any part of its body.

### CASE III.

Rebecca Gill, aged thirty-one, in the ninth month of pregnancy, was inoculated June 6th. On the 13th the eruptive fever commenced, and on the 16th the eruption appeared. On the 18th ſhe was delivered of a living child, without any appearance of diſeaſe upon it.

### CASE IV.

M. Twining, aged thirty-eight, was inoculated July 1ſt, in the fifth month of pregnancy. She had the diſeaſe favourably, and was delivered of a living child at the end of the ninth month, without any appearance of the diſeaſe upon it.

All the patients were inoculated without any previous preparation; but the following evening took a powder of calomel and emetic tartar, and the next morning a doſe of Glauber's ſalt. Nothing more was adminiſtered till the eruptive fever commenced; nor even then, unleſs the ſymptoms ran high, in which caſe a powder, compoſed of calomel and jalap, was given, which generally reduced the febrile heat, and leſſened the number of the puſtules.

The

The women and children in the lying-in month had the difeafe mildly. Dentition aggravated the fymptoms; but the danger was obviated by purgatives and the ufe of the gum lancet. The patients who were reduced by the malignant fever had but few puftules, and thofe did not arrive at a ftate of maturation without the affiftance of bark and wine. The eruptive fever in thofe who were labouring under intermittents ufually commenced with a violent pain in the back, which was commonly relieved by a proper dofe of *tinct. thebaic* and *vin antimon.* after a ftool or two had been procured. The puftules were few in number; and on the recovery of the patients from the fmall-pox, the intermittents left them.

Dr. Cullen obferves, that "it merits inqui-"ry, whether every difeafed ftate fhould re-"ftrain us from the practice of inoculation, "or what are the particular difeafes that fhould "do fo * ?"—If we may judge from the preceding obfervations, it will appear that there are few difeafes in which we may not with fafety venture to inoculate, even under the difadvan-

* Firft Lines of the Practice of Phyfic, Vol. II. p. 150. 4th edition.

tages of ſeaſon, a proper attention being paid to the diſeaſe that previouſly exiſted; and that when a diſeaſed perſon is in danger of receiving the infection, it will be more adviſeable to inoculate the patient, let the diſeaſe be what it will, than run the hazard of his receiving the infection in the natural way. Even during pregnancy, which, till lately, has been conſidered as a ſtate highly dangerous to receive the infection in, (and I have lately ſeen many inſtances in which it was ſo in the natural way) experience has ſhewn that inoculation may be practiſed with ſafety.

I obſerved that the number of puſtules was in proportion to the height of the eruptive fever, and conclude from thence that every diſeaſe that has a tendency to induce or heighten a febrile ſtate muſt be attended with danger in proportion to its tendency thereto.

*Painſwick,*
*Glouceſterſhire,*
April 5, 1786.

XI. *Obſer-*

